# Efficacy and safety of acupuncture in combination with Chinese herbal medicine in dealing with osteoporosis: A protocol for a systematic review and network meta-analysis

**DOI:** 10.1097/MD.0000000000032441

**Published:** 2022-12-30

**Authors:** Piao Long, Shicong Ju, Jun Wang

**Affiliations:** a College of Traditional Chinese Medicine, Hunan University of Chinese Medicine, Changsha, China; b Acupuncture Department, The First Hospital of Hunan University of Chinese Medicine, Changsha, China.

**Keywords:** acupuncture, Chinese herbal medicine, network meta-analysis, osteoporosis, protocol, systematic review

## Abstract

Osteoporosis (OP) has emerged as a major global public health issue due to its high prevalence, unknown pathogenesis, and lack of specific drugs for prevention and treatment. Studies have demonstrated that acupuncture in combination with Chinese herbal medicine (CHM) is effective in treating OP. However, there is a scarcity of experience and high-quality evidence. A network meta-analysis and systematic review were used to evaluate the efficacy and safety of acupuncture in combination with CHM for the treatment of OP.

**Methods:**

Comprehensive search of Chinese databases such as China National Knowledge Infrastructure, VIP, Wanfang, China Biomedical Literature Database and English databases for example PubMed, Cochrane Library, EMbase, etc. The search period was extended from the creation of the database to November 2022. All randomized controlled trials on acupuncture in combination with CHM in dealing with OP were collected. After literature analysis and data extraction, the Cochrance system was used to evaluate the high quality of the included articles and Stata 14.0 was used for the network meta-analysis.

**Results:**

The current systematic review and network meta-analysis will provide the effectiveness and safety of acupuncture in combination with CHM in dealing with OP.

**Conclusion:**

The research will provide reliable evidence for the clinical use of acupuncture in combination with CHM in dealing with OP.

## 1. Introduction

Osteoporosis (OP) is a systemic metabolic bone disease caused by multiple causes, with a high prevalence, unknown pathogenesis, and lack of specific preventive and therapeutic drugs, and thus has become an important global public health problem. The incidence of OP is highly correlated with age.^[1]^ In few years, the occurrence of OP has increased significantly as the world population has aged, with approximately 200 million OP patients worldwide.^[2]^ At the same time, OP patients are very prone to fragility fractures – osteoporotic fractures, which can cause complications such as infection and thrombosis, and even lead to disability and death of patients, and has a negative effect on patients’ health.^[3,4]^ Disturbances in the homeostasis of bone remodeling are the underlying cause of OP. Osteoblasts create new bone during the physiological process of bone remodeling, while osteoclasts resorb the existing bone matrix. which is a key process in maintaining healthy bone tissue in adults, and multiple factors are involved in regulating this process.^[5]^ Bisphosphonates, estrogens, and raloxifene are commonly used to treat OP by reducing the number of osteoclasts, inhibiting bone resorption, slowing bone loss, and maintaining bone health.^[6]^ Although these drugs significantly increase bone mass, they have limitations and side effects, such as suboptimal efficacy in a large number of patients, thromboembolism and gastrointestinal irritation.^[7,8]^ Therefore, it is necessary to find new drugs to improve OP while minimizing side effects.

Acupuncture and Chinese herbal medicine (CHM) are important components of Chinese traditional medicine. The most significant one is non-pharmacological treatments in Chinese medicine is acupuncture, and it entails using metal needles and piercing the patient’s acupuncture points at a specific angle to stimulate specific parts of the body, thus treating the disease. Acupuncture becomes the clinical treatment of OP because it is inexpensive, effective, green, and noninvasive, and studies have shown that acupuncture is one of the effective therapies for the treatment of OP.^[9,10]^ Because of its abundant resources, ideal efficacy, and low adverse effects, CHM has made significant progress in the treatment of OP.^[11]^

The combination of acupuncture therapy and CHM for OP can provide clinical relief by relieving pain, improving bone mass, reducing fracture risk, and ultimately improving patients’ quality of life.^[12]^ The combination therapy has good systemic modulation effects and few adverse effects occur, providing a broad market for the treatment of OP.^[13]^ Although some RCTs have studied acupuncture and CHM as both effective and safe in OP, there are problems of small sample size and insufficient methodological quality, and its efficacy and safety lack systematically evaluated. Therefore, in this study, we searched the published randomized controlled trials (RCT) literature of acupuncture therapy and CHM for OP, and observed its efficacy, bone mineral density, serum calcium, serum sclerostin, adverse reactions and other data, and analyzed the data of its efficacy, bone mineral density, serum calcium, and adverse reactions. The research will provide reliable evidence for the clinical use of acupuncture in combination with CHM in dealing with OP.

## 2. Methods

### 2.1. Registration

The protocol for this systematic review and network meta-analysis is based on the PRISMA-P (Preferred Reporting Items for Systematic Reviews and Meta-Analyses) protocols.^[14]^ This protocol is registered with PROSPERO (registration number: CRD42022377217).

### 2.2. Ethics

Ethical approval was not necessary, this study does not involve animal welfare.

### 2.3. Inclusion criteria

#### 2.3.1. Research types.

This study collect all RCTs on acupuncture in combination with CHM for OP patients. It would be only in Chinese and English, regardless of the blindness, publication status, or location.

#### 2.3.2. Research objects.

For patients with a definite diagnosis of OP, there are no limitations in terms of nationality, race, gender, age, occupation, duration, and onset of the disease.

#### 2.3.3. Intervention measures.

The control group received acupuncture or CHM treatment, and the treatment group received acupuncture in combination with CHM. There are no restrictions on the dosage form, dose or duration of CHM.

#### 2.3.4. Outcome indicators.

The main indicator was the overall effective clinical effectiveness rate, and the secondary indicators were lumbar spine and femoral neck bone mineral density, 1,25-dihydroxyvitamin D3, alkaline phosphatase, serum phosphorus, serum calcium, and adverse drug reactions.

### 2.4. Exclusion criteria

In the process of treating OP, the intervention measures did not use acupuncture combined with CHM randomized controlled trials, non-randomized controlled trials, relevant animal experiments, reviews, abstracts, conference-type literature, case reports, duplicate publications and articles without data information.

### 2.5. Retrieval strategy

Electronic computer search Chinese literature is mainly for China Biomedical Literature Database, China National Knowledge Infrastructure (CNKI), VIP, and Wanfang. English literatures were searched through PubMed, EMbase, and Cochrane Library. The words “acupuncture”, “Chinese herbal medicine”, “osteoporosis” and their synonyms were used as search terms, and the search was conducted from database creation to November 2022. The search strategy, following the example of PubMed, is shown in Table 1.

**Table 1:** PubMed database search strategy.

Number	Search terms
#1	“osteoporosis”[Mesh] OR “OP”[Ti/Ab] OR “senile osteoporosis”[Ti/Ab] OR “osteoporosiss, senile”[Ti/Ab] OR “postmenopausal osteoporosis”[Ti/Ab] OR “osteoporosis, postmenopausal” [Ti/Ab] OR “age-related osteoporosis ” [Ti/Ab] OR “bone losse, postmenopausal”[Ti/Ab] OR “postmenopausal bone losses ”[Ti/Ab]
#2	“acupuncture”[Mesh] OR “acupuncture therapy”[Ti/Ab] OR “acupuncture treatment”[Ti/Ab] OR “point”[Ti/Ab] OR “needle”[Ti/Ab] OR “warm needling” [Ti/Ab] OR “acusector”[Ti/Ab] OR “gastric acupuncture”[Ti/Ab] OR “skin acupuncture”[Ti/Ab] OR “wrist-ankle acupuncture”[Ti/Ab]
#3	“Chinese herbal medicine”[Ti/Ab] OR “CHM”[Ti/Ab] OR “traditional Chinese medicine”[Ti/Ab] OR “TCM”[Ti/Ab] OR “herbals”[Ti/Ab] OR “herbal medicine” [Ti/Ab] OR “Chinese Medicine” [Ti/Ab]
#4	#2 AND #3
#5	“randomized controlled trial”[Mesh] OR “RCT”[Mesh] OR “randomly”[Ti/Ab] OR “randomized” [Ti/Ab] OR “controlled clinical trial”[Ti/Ab] OR “clinical trial”[Ti/Ab]
#6	#1 AND #4 AND #5

### 2.6. Literature selection and data collection

**Figure 1.**
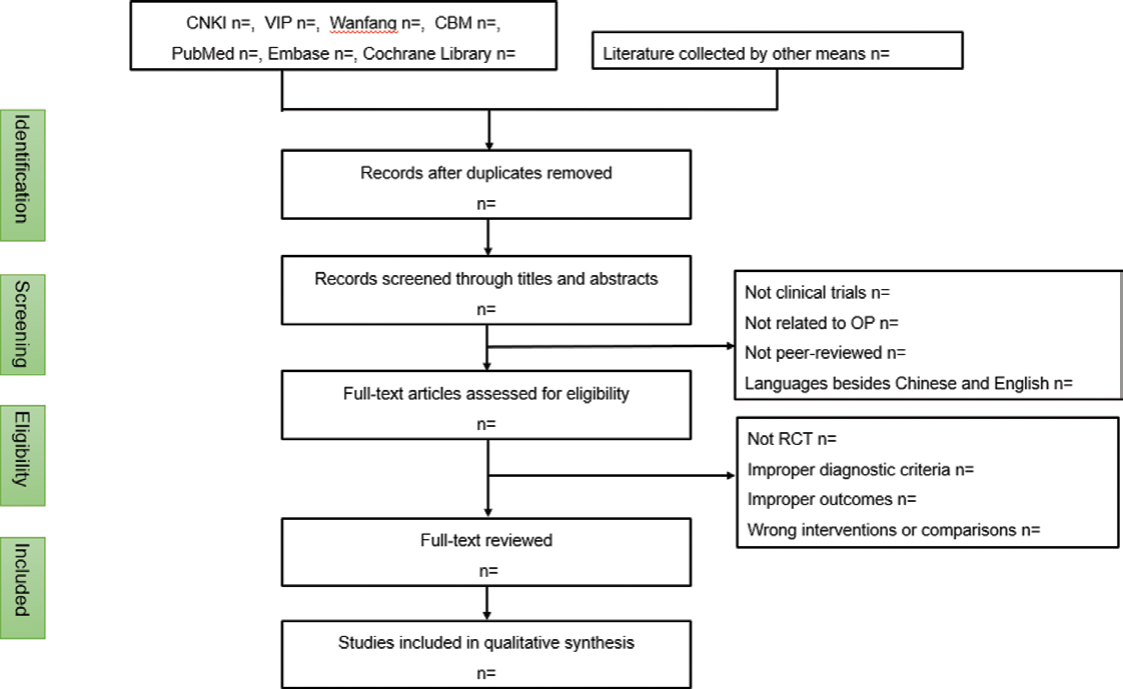
PRISMA flow diagram of selection process. PRISMA = Preferred Reporting Items for Systematic Reviews and Meta-Analyses.

Two researchers examined the literature independently of each other and extracted data that met the inclusion criteria, including the patient information, interventions, and outcome measures were judged by the third investigator in case of disagreement. A PRISMA flow diagram is created that describes the entire selection process (Fig. 1).

### 2.7. Evaluation of literature quality

The high quality of the literature was evaluated according to the Cochrane 5.0.1 Manual of Systematic Review, from the 6 aspects of randomization method, hidden attribution, blinding, integrity of results, selective reporting of research findings, and other factors that may potentially affect the authenticity. The quality of the literature was evaluated at 3 levels: “low” (low bias), “unclear” (uncertain bias or lack of relevant information) and “high” (low bias).

### 2.8. Network meta-analysis

Performing a network meta-analysis relies on the Bayesian model, merge and compare the direct evidence and indirect evidence of the included studies, use R4.0.2 software and Ge MTC to set the chain and number of iterations. The Brooks-GeIman Rubin diagnostic method was used to judge the degree of model convergence, that is, the mean value of the reduction factor after iteration and 97.5% tended toward 1 and reached stability after iterative calculation, indicating that the degree of model convergence was satisfactory. In addition, Stata 16.0 software was used to calculate and draw the surface under the cumulative ranking (SUCRA) to intuitively reflect the relative pros and cons of efficacy and safety between drugs. SUCRA values range from 0 to 1, with higher SUCRA values signifying better efficiency.

### 2.9. Assessment of heterogeneity

Heterogeneity tests were performed on studies, with the effect model using *I*^2^ = 50% as the critical value and *I*^2^ greater than 50% suggesting the existence of large heterogeneity.

### 2.10. Publication bias

Use Stata 16.0 to draw comparison-adjusted funnel plots, and conduct publication bias analysis according to whether the funnel plot is symmetrical and the results of Egger and Begg tests. The funnel plot should be symmetrical along the midline around the regression line, with a *P* value > .05 for Egger, Begg’s test.

### 2.11. Sensitivity evaluation

Sensitivity analysis was performed on the inclusion indicators, the result of the network Meta-analysis, in which a certain RCT was excluded one by one, were unchanged, which proved that the results of meta-analysis were relatively stable.

## 3. Discussion

OP is a common orthopedic disease characterized by degeneration of bone tissue microarchitecture, low bone mass, and reduced bone strength. With the aging population, the number of OP cases is increasing every year.^[1]^ With the progression of the disease, the density and strength of bone tissue continue to decline, and patients have low back and even systemic bone pain, and the risk of fracture increases.^[15]^ Chinese traditional medicine has a rich history of being used for OP, with acupuncture and CHM being highly effective. Acupuncture therapy and CHM are widely applied to clinical management of OP as an indispensable part of Chinese traditional medicine, and their effectiveness has been recognized by a large number of patients and physicians, gradually gaining the attention of researchers at home and abroad. Acupuncture in conjunction with CHM for OP can alleviate symptoms, improve clinical efficacy, and promote functional recovery.

In this study, we searched published RCT literature on acupuncture in combination with CHM in dealing with OP, collected relevant data for systematic review and network Meta-analysis, in order to provide evidence-based medical evidence for acupuncture combined with CHM for OP. However, a comprehensive quantitative analysis could not be carried out due to different clinical diagnosis and evaluation criteria for clinical symptoms, biased literature publication, and short follow-up time. Therefore, it is necessary to conduct further RCT experiments with high quality, large sample size and long follow-up time to confirm the efficacy and safety of acupuncture in combination with CHM for OP.

## Author contributions

**Conceptualization:** Piao Long.

**Data curation:** Piao Long.

**Formal analysis:** Shicong Ju.

**Funding acquisition:** Jun Wang.

**Investigation:** Shicong Ju.

**Methodology:** Shicong Ju.

**Software:** Piao Long, Shicong Ju.

**Supervision:** Jun Wang.

**Validation:** Piao Long, Jun Wang.

**Writing – original draft:** Piao Long.

**Writing – review & editing:** Jun Wang.
